# P-2247. Comparison of a Cellular Host-Response Test to Blood Culture Results for Risk Stratification and Diagnosis of Sepsis

**DOI:** 10.1093/ofid/ofae631.2400

**Published:** 2025-01-29

**Authors:** Matt Sorrells, Roya Sheybani, Ajay M Shah, Hollis R O’Neal, Robert Scoggins

**Affiliations:** Cytovale Inc, San Francisco, California; CytoVale, Inc., San Francisco, California; CytoVale, Inc., San Francisco, California; Louisiana State University Health Sciences Center, Baton Rouge, Louisiana; Cytovale Inc., San Francisco, California

## Abstract

**Background:**

Sepsis is a life-threatening syndrome that requires urgent intervention to achieve optimal outcomes[1-3]. While considered the gold standard for bacteremia and sepsis diagnosis, blood cultures (BCX) require long turnaround and offer low sensitivity [4], with only 18-25% of patients with sepsis returning positive BCXs [5]. In this study, we compared a cellular host response test to BCX in performance for diagnosing and risk-stratifying patients suspected of sepsis in the ED.

Figure 1

**Figure:**
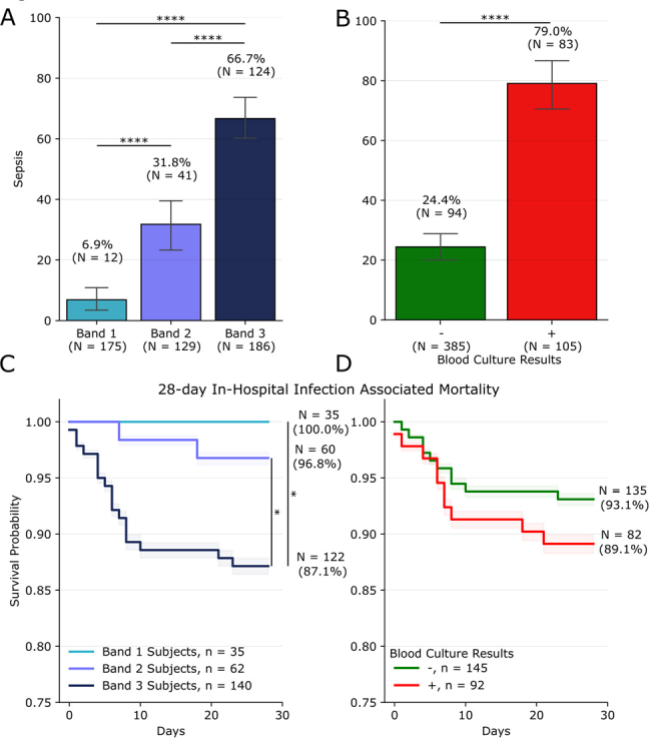

Rate of sepsis status, per Sepsis-3 definition, across HR interpretation bands (A) and blood culture results (B). 28-day survival probability for in-hospital infection associated mortality for (C) HR interpretation bands and (D) blood culture results. * and **** represent p < 0.05 and p < 0.0001, respectively.

**Methods:**

A semi-quantitative in-vitro cellular host-response test (HR) uses deformability cytometry to assess leukocyte biophysical properties from whole blood in < 10 min. The test generates an Index based on increasing WBC deformability, stratified into 3 interpretation bands (Band 1, Band 2, Band 3) of increasing sepsis likelihood [6].

Adult patients presenting to the ED with signs or suspicion of infection were prospectively enrolled at multiple US sites (02/2016–10/2021). EDTA-anticoagulated blood was assayed 5 hours from draw using the test, and patients were followed by retrospective chart review. Infection and sepsis status was determined via retrospective physician adjudication per Sepsis-3, during which adjudicators were blinded to HR results but had BCX results visible.

Table 1

**Figure:**
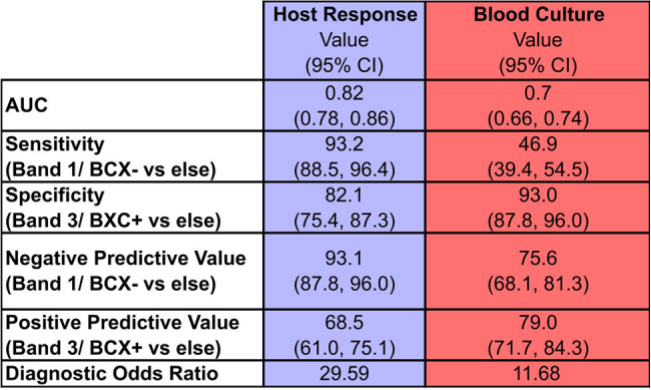

Sepsis-3 receiver operator characteristic diagnostic performance for HR compared to blood culture results.

**Results:**

Our cohort of 490 patients for whom BCXs were ordered was stratified into 175 Band 1, 129 Band 2, and 186 Band 3 patients for HR versus 385 BCX+ and 105 BCX- patients. When evaluated for diagnostic performance for Sepsis-3, HR, compared to BCX, yielded higher AUC (0.82 [0.78 - 0.86 95% CI] HR vs. 0.7 [0.66 - 0.74] BCX), higher sensitivity (93.2% HR vs 46.9% BCX), and higher negative predictive value (93.1% HR vs 75.6% BCX) with similar specificity (82.1% [75.4 - 87.3%] HR vs 93.0% [87.8 - 96.0%] BCX) and positive predictive value (68.5% [61.0 - 75.1%] HR vs 79.0% [71.4 - 84.3%] BCX,Fig. 1 A/B, Tab. 1). For infection-associated mortality, survival rates of 100% (N = 35 surv. /35 inf.) and 87.1% (N = 122 surv. /140 inf.) were found for Bands 1 & 3, respectively (p < 0.05) compared 93.1% (N = 135 surv. / 145 inf.) and 89.1% (N = 82 survived /92 infected) for BCX - & +, respectively (p > 0.05).

References

**Figure:**
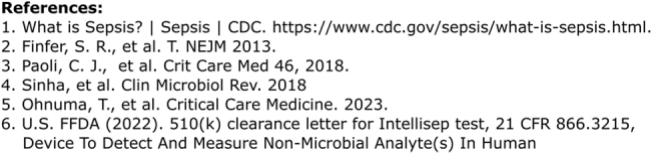

**Conclusion:**

These findings indicate that the HR test may provide more accurate and timely results for sepsis diagnosis and risk stratification when compared to BCX.

**Disclosures:**

Matt Sorrells, PhD, Cytovale Inc.: Employee Roya Sheybani, PhD, Cytovale Inc.: Employee Ajay M. Shah, PhD, Cytovale Inc.: CEO Robert Scoggins, MD, PhD, Cytovale Inc.: Employee, CMO

